# Spalding’s Porcelain Jacket Crown

**Published:** 1904-05

**Authors:** Henry W. Gillett

**Affiliations:** New York


### SPALDING'S PORCELAIN JACKET CROWN.[1]

BY HENRY W. GILLETT, D.M.D., NEW YORK.

FOLLOWING the suggestion of Dr. C. H. Land for a porcelain jacket crown, Dr. Edward B. Spalding, of Detroit, has worked out a different technique for a crown, which is so artistic in final result, and so fascinating in the precision of the different steps of its development, that it arouses immediate interest wherever shown.

Most of you are probably familiar with the article by Dr. Land in the *Dental Cosmos* for August, 1903, on "Porcelain Dental Art," in which he figures a porcelain crown similar to the one I will show you to-night.

Dr. Spalding wishes to give credit to Dr. Land for the original suggestion of such a crown. The development of the technique of the crown I am to show you is entirely Dr. Spalding's work, and seems to me a distinct advance over Dr. Land's.

In order to describe intelligibly Dr. Spalding's procedure, we will assume that we are dealing with a deformed superior cuspid tooth, with either living or dead pulp, but with such defects of development as to call for treatment of some kind. The first step is to take a wire measurement of the circumference of the tooth at the gum margin. The second is to give the crown of the tooth a conical shape from the gum margin to the occlusal end, to give

---

[1] Read before The New York Institute of Stomatology, January 5, 1904.

it a distinct shoulder at or just under the gum margin, and to shorten it. This Dr. Spalding accomplishes with rather thin three-quarter-inch disks of carborundum for the broad surfaces, and finishes with small inverted cone carborundum points, mounted for both hand-pieces, at the angles resulting from the use of the larger disk.

The third step is to cut a piece of matrix platinum in the shape of a trapezoid, with its base line the same length as the wire measurement in the first step.

Fourth, unite the edges of this piece with a minimum quantity of pure gold, forming a truncated cone-shape. Slip this over the tooth, making sure that its edge goes above the shoulder left at the gum margin, and with the dentimeter twist a soft wire ligature around the matrix just below the shoulder, and with suitable pliers begin to draw the surplus platinum to the mesial and distal sides in two flattened wings, and also over the end of the tooth. Clip off most of the surplus at the end, leaving a little for a handle till the fitting is nearly done, and fold over the surplus at the sides, cutting off part of it first if there is much excess. Burnish the matrix accurately and smoothly to the tooth. Dr. Spalding uses for this work a bone instrument, of which I have made a rough imitation for your inspection. It is a valuable instrument, and my use of it convinces me that a bone or ivory burnisher will be very helpful in inlay matrix formation. The feel of the bone instrument as it passes over the platinum is a distinct relief as compared with the drag of a steel instrument.

Fifth, select a rubber tooth (Dr. Spalding uses at present Consolidated teeth) of suitable color and shape, and with carborundum stones cut it to the shape of the sample facing shown. Note that the tip is hollowed out and the shoulder and enamel surface of the palatal side of the tip is left intact. This is accomplished by using the same small carborundum stone used for finishing the shaping of the tooth. This shoulder is a very important aid in the further work.

Sixth, wax this to the matrix, with latter in correct position on the tooth, using gutta-percha and wax mixture; remove carefully; grip with suitable pliers, one jaw of which passes inside the matrix and the other engages a corresponding point on the labial surface of the facing. Remove the wax and place "block body" on one side near the tip and jar it carefully through till it appears on the other side, then build it round the top of the cone and, setting on end in the furnace, fuse the body. It is essential that the body for this first bake be kept away from the shoulder in the matrix. This block body Dr. Spalding makes by grinding up in the mortar pinless teeth of the same make as the facing he uses.

Seventh, place the piece in position on the tooth and carefully perfect the fit of the matrix at the shoulder.

Eighth, apply body, carve, and fuse to finish.

Ninth, remove the matrix and set in suitable cement.

Dr. Spalding is able to report use of these crowns only since last April,—about nine months. This is, of course, too short a period to enable us to define their permanent place in our resources for such work, but his report for that time is favorable without exception. He has had no breakages yet. He has the matter so thoroughly worked out, and is so conservative in his own estimate of it, that the fact that he continues to think well of the method is sufficient evidence that it has value.

For molars, Dr. Spalding builds up the entire crown with block body.

A possible objection to these crowns over live pulps is the difficulty of getting access to the pulp in case pulpitis develops after the application of the crown.

